# Cognition in Sensorimotor Control: Interfacing With the Posterior Parietal Cortex

**DOI:** 10.3389/fnins.2019.00140

**Published:** 2019-02-27

**Authors:** Srinivas Chivukula, Matiar Jafari, Tyson Aflalo, Nicholas Au Yong, Nader Pouratian

**Affiliations:** ^1^Division of Biology and Biological Engineering, California Institute of Technology, Pasadena, CA, United States; ^2^Department of Neurological Surgery, Los Angeles Medical Center, University of California, Los Angeles, Los Angeles, CA, United States

**Keywords:** posterior parietal cortex, peripersonal space, PPC, neuroprosthetics, motor, decision making, coordinate transformations

## Abstract

Millions of people worldwide are afflicted with paralysis from a disruption of neural pathways between the brain and the muscles. Because their cortical architecture is often preserved, these patients are able to plan movements despite an inability to execute them. In such people, brain machine interfaces have great potential to restore lost function through neuroprosthetic devices, circumventing dysfunctional corticospinal circuitry. These devices have typically derived control signals from the motor cortex (M1) which provides information highly correlated with desired movement trajectories. However, sensorimotor control simultaneously engages multiple cognitive processes such as intent, state estimation, decision making, and the integration of multisensory feedback. As such, cortical association regions upstream of M1 such as the posterior parietal cortex (PPC) that are involved in higher order behaviors such as planning and learning, rather than in encoding movement itself, may enable enhanced, cognitive control of neuroprosthetics, termed cognitive neural prosthetics (CNPs). We illustrate in this review, through a small sampling, the cognitive functions encoded in the PPC and discuss their neural representation in the context of their relevance to motor neuroprosthetics. We aim to highlight through examples a role for cortical signals from the PPC in developing CNPs, and to inspire future avenues for exploration in their research and development.

## Introduction

Motor neuroprosthetics are applications of brain-machine interfaces (BMIs) that acquire brain signals, decode them, and translate them into commands that are relayed to effectors to carry out desired actions (Andersen et al., 2010). A primary goal for such systems is to augment or to restore lost function to people with devastating neurological injury (such as spinal cord injury) (Hochberg et al., 2006; Pesaran et al., 2006a; Bouton et al., 2016). Cortical signals for these devices have typically been derived from the primary motor cortex (M1). This has the advantage of being close to the motor output and providing information highly correlated with desired movement trajectories (Quian Quiroga et al., 2006). However, coordinated dynamics are a complex interplay of movement intent with state estimates and sensory feedback. As such, areas upstream of M1 such as the posterior parietal cortex (PPC) that are involved both in sensory processing and in motor planning, rather than in encoding movement itself, may enable enhanced, cognitive control of neuroprosthetics. These are termed cognitive neural prosthetics (CNPs) (Andersen and Buneo, 2002; Musallam et al., 2004; Andersen et al., 2014).

The PPC is a cortical association region near the intraparietal sulcus (IPS) that receives multisensory input and interfaces at its rostral end with motor networks. It is thus uniquely situated for multisensory integration. BMI research in non-human primates (NHPs) and in human subjects has confirmed that the PPC carries representations of space, spatial awareness, perceptual decision making, contextual memory, learning as well as motor planning (Andersen et al., 1987; Snyder et al., 1998; Andersen and Buneo, 2002). A growing number of single-neuron studies of the PPC has contributed to a greater understanding of the neural basis for these representations as well as to an understanding of the complex computational transformations that are performed within the PPC to act upon the spatial information encoded by the various sensory modalities in different reference frames (Bremner and Andersen, 2014). It is apparent that each of these dimensions of neural encoding may be exploited to implement the most intuitive and versatile CNPs.

In this review, we first provide a broad overview of the functional organization of the PPC. Next, we illustrate, through a small sampling, cognitive functions encoded in the PPC and discuss their neural representation in the context of their relevance to motor neuroprosthetics. Because the availability of single neuron data from humans is still scarce, most research presented is from NHP experimentation unless otherwise specified. We do not aim through this brief review to summarize the volumes of exemplary research to date, but rather to highlight a role for cortical signals from the PPC in developing CNPs and to inspire future avenues for exploration in their research and development.

## Anatomical and Functional Organization of the Posterior Parietal Cortex

**FIGURE 1:**
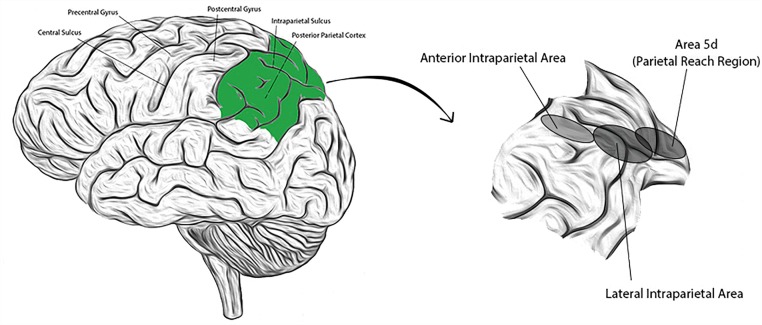
**(Left)** Lateral view of the human brain with identifying landmarks (the posterior parietal cortex is colored green); **(Right)** the posterior parietal cortex is expanded and the approximate locations of three labeled sub-regions indicated by the corresponding, shaded ellipses.

The PPC is comprised of anatomically distinct sub-regions that collectively receive and integrate multisensory input (visual, auditory, somatosensory, and vestibular), enabling a representation of higher level, cognitive functions. Regions have been identified (primarily from NHP studies) with high concentrations of neurons responsive to saccades and perceptual decision making (lateral intraparietal area; LIP), reaching movements (parietal reach region; PRR, and within this region, area 5d), grasping movements (anterior intraparietal area; AIP), or representations of the body and its surroundings (area 5d, and in NHPs, the ventral intraparietal area; VIP) (Mountcastle et al., 1975; Murata et al., 2000; Beurze et al., 2009; Hwang and Andersen, 2013). Presumed homologs to these areas have been identified in humans and their approximate locations are shown in Figure 1 (Culham et al., 2006; Seelke et al., 2012; Sereno and Huang, 2014). Recent single neuron studies in human subjects suggest that cortical areas may also demonstrate functional segregation (in contrast to the anatomic segregation described in NHPs) (Zhang et al., 2017). Experience in humans is limited, however. We introduce below three PPC sub-regions extensively studied in NHPs that will be revisited in subsequent sections.

### Lateral Intraparietal Area (LIP)

The LIP is located on the lateral bank in the middle of the IPS, and predominantly encodes spatially selective, eye movement related activity (Snyder et al., 1998). It is a primary site for learned categorization of various attributes of visual stimuli, including motion direction and shape (Freedman and Assad, 2006). It carries representations of perceptual decision making (in the context of responding with spatially preferred saccades), working memory, and the timing of upcoming goal-directed eye movements (Gnadt and Andersen, 1988; de Lafuente et al., 2015). Goal locations in LIP neurons are encoded in eye-centered coordinates and have been used to improve decoding of reach targets when combined with neural activity from other PPC regions (such as PRR) (Cohen and Andersen, 2002).

### Anterior Intraparietal Area (AIP)

The AIP is located in the anterior aspect of the IPS at its junction with the postcentral sulcus, and contains neurons responsive primarily to objects and the hand postures to grasp them (Baumann et al., 2009; Lehmann and Scherberger, 2013). Inactivation of AIP in NHPs hinders coordinated finger motion during grasping movements (Gallese et al., 1994). In human experiments, single neurons from the AIP have been found to be selective for a variety of imagined hand postures (even independent of graspable objects) that can be used to control artificial prosthetic hands (Klaes et al., 2015). Small numbers of AIP neurons have been found sufficient to decode a repertoire of intention-based grasp postures, which are encoded within these neurons in a hand centered reference frame (Murata et al., 2000; Klaes et al., 2015).

### Area 5d (and Macaque Ventral Intraparietal Area; VIP)

Area 5d, a part of the PRR, is located on the posterior surface of the superior parietal lobule, abutting the IPS. It carries signals related both to movement goals and trajectories, and encodes target location predominantly in hand centered coordinates (Bremner and Andersen, 2012). Similar to the VIP in macaques, area 5d also carries a high concentration of multimodal (especially bimodal) neurons with receptive fields (RFs) to multisensory stimuli aligned in a congruent, body part centered manner (Avillac et al., 2005). This may relate to the contiguous nature of the VIP with area 5d, from gyrus into the IPS; neurons in this region may form a substrate to represent similar processes along a Such cells are believed to be involved in the construction of a multisensory representation of body schema and surrounding space, and may also form the basis for the interpretation of actions performed by, or to, others (represented in so called “mirror neurons”) (Rizzolatti et al., 2014).

## Reference Frames

Our brain performs a remarkable series of coordinate transformations in allowing a smooth interaction with the environment (Andersen and Cui, 2009). In reaching for a cup, for instance, the brain determines appropriate axes to represent in space the configuration of the arm (effector) and the position of the handle (object) to bring one to the other. Understanding the coordinate transformations that the brain intrinsically computes in the process of motor control is essential in eventually utilizing cortical neural signals for BMI applications. Single neuron data from both humans and NHPs suggest that spatial locations are represented in multiple reference frames that can vary within and across sensory modalities (Avillac et al., 2005). For instance, visual stimuli can be encoded with respect to the eye or the head, and tactile stimuli may likewise be encoded with respect to the eyes or body parts (e.g., hands) (Buneo et al., 2002; Pesaran et al., 2006b, 2010). There appear to be anatomically defined, regional preferences for the coordinate frames utilized. Both visual and auditory stimuli are encoded in a common gaze-centered reference frame by neurons in most regions of the PRR, but in hand-centered coordinates by area 5d (Andersen and Cui, 2009; Crawford et al., 2011; Bremner and Andersen, 2012). Moreover, reference frames can dynamically evolve even in the same population of neurons with changing behavioral demands, ensuring that the most relevant postural and spatial information is encoded at each behavioral stage (Chang et al., 2009; Chang and Snyder, 2010; Bremner and Andersen, 2014).

The process of converting sensory stimuli into motor commands (sensorimotor transformation) has historically been thought to occur through the remapping of multiple modalities into a common reference frame (Avillac et al., 2005). Neurons in distinct brain areas were predicted to have RFs with positions invariant of reference frame (Duhamel et al., 1997). However, multiple multisensory areas (such as the superior colliculus) demonstrate partially shifting RFs (Trotter and Celebrini, 1999; Pouget and Snyder, 2000). For instance, in the superior colliculus, eye centered locations of a RF change with eye movement, but by less than the change in eye position, and similarly for auditory RFs for the same neural units (Pouget et al., 2002). Moreover, the amplitude of the neural response (the height of the RF) may be modulated by eye position, creating a phenomenon referred to as a “gain field” (Andersen et al., 1985; Pouget and Snyder, 2000). Many neural units may in fact be “basis function units” – cells that represent (through nonlinear functions) the collective sensory input from multiple modalities in various reference frames (Pouget et al., 2002). A mixture of reference frames in such cells, which appear to be heterogeneously distributed around the brain, may explain partial shifts in RFs, and gain fields (Pouget et al., 2002).

“Mixed selectivity” is a relatively recent concept that is increasingly recognized as fundamental to the coding and implementation of brain functions (Rigotti et al., 2013). It refers to the idea that neurons behave differently in different contexts, belonging to different ensembles that encode explicit but highly context-dependent information (Fusi et al., 2016). The common neural substrate may provide a basis for the transfer of learning: motor skills acquired through one ensemble may be recruited by others (Zhang et al., 2017). This provides an alternative viewpoint for gain fields and multiple reference frames; a single neuronal population may encode many types of information in a context dependent manner. A direct consequence of this is that overlapping activity at the population level may afford the brain significant computational savings through shared resources, such as different views of the object, different starting configurations of the effector, even information from different brain areas (Fusi et al., 2016; Zhang et al., 2017). Within the framework of mixed selectivity, neurons can greatly enhance the diversity, and dimensionality of neural representations that can be encoded by a subset of neurons (Rigotti et al., 2013). This has important implications for cortical neuroprosthetics, foremost amongst them that large volumes of data may be extracted from cortical signals derived from strategic neuronal applications. The rich, higher level, cognitive processing encoded within the PPC appears to suggest that it may serve as one such cortical brain region. A practical example would be that within the PPC, small neuronal groups appear to encode the rich dynamic interplay involved in complex movements such as identifying a cup on a table, reaching for it and bringing it to the mouth to drink from it. A decoder trained on PPC neural signals, especially within this context, could benefit from simultaneously enabling the coordinated visual and gross motor coordination. This would be a large leap forward from learning the individual basis functions of neurons within context specific behaviors in lower order brain regions (non-association regions), and from there deriving more complex motor computations, thereby limiting error interference, reducing redundancy and conferring upon evolving neural networks and machine learning algorithms a quicker adaptability to new contexts or to new tasks.

## Movement Related Decision Making

In daily life, motor actions are dictated by environmental cues that often occur simultaneously. Decision processes must consider the saliency of all stimuli, and appropriately direct attention and action. The PPC plays a fundamental role in the cognitive, movement related decision making that underlies its representation of motor intentions, such as for eye and limb movements (in the LIP and PRR, respectively) (Gottlieb et al., 1998; Gottlieb, 2017). This is a complex process influenced by contextual attention and awareness, and involves a rapid integration of values assigned to available sensory evidence with expected action reward and memory of previous action outcomes (Dorris and Glimcher, 2004; Desmurget and Sirigu, 2009; de Lafuente et al., 2015). Within the PPC, these multiple dimensions significantly modulate the encoding and processing of one another (Peck et al., 2009; Foley et al., 2017). For instance, LIP neurons encode salient visual stimuli within preferred visual fields but their attentional bias is influenced by the association between the perceived stimulus and the expected value of an action (Mevorach et al., 2006; Gottlieb, 2017). Moreover, they have been found to encode a memory of prediction error which is incorporated into shifts in attention and influences subsequent action selection. This suggests that the PPC plays an active role in the combinatorial decision-making processes of motor behaviors.

To the extent that making good decisions requires choosing relevant information from competing distractors, PPC (LIP) neurons encode the reduction in sensory variability that a movement (saccade) is expected to bring (Corbetta et al., 2000; Friedman-hill et al., 2003). Similar representations for hand reaching actions have been found within areas of the PRR (such as the macaque medial intraparietal area; MIP) (Gnadt and Andersen, 1988; Snyder et al., 1997). Remarkably, though, the regional functionality is also distinct: NHP studies have demonstrated impaired free choice decisions for reaches but not for saccades, with PRR inactivation (Christopoulos et al., 2015). In other human studies, transcranial magnetic stimulation (TMS) of the right and left PPC in human subjects resulted in biased saccades toward and away from salient environmental stimuli, respectively (Friedman-hill et al., 2003). Taken together, these studies suggest that the PPC contributes to filtering sensory distractors and focusing attention on behaviorally relevant stimuli in a top-down fashion, but possibly in an effector-specific manner.

In addition to evaluating saliency and encoding contextual action value, PPC neural networks also appear to provide a memory substrate for history-dependent action selection (Morcos and Harvey, 2016). Through two-photon calcium imaging and optogenetic inactivation studies in mice, it was recently demonstrated that PPC neurons encode a history of action choice as it relates to their outcomes, and that the history-dependency of motor decision making diminishes with PPC inactivation (Hwang et al., 2017). Because PPC neurons exhibit selective tuning (for example, spatial tuning for preferred saccade directions in LIP neurons), different sets of PPC neurons maintain different representations of choice-outcome history (different values for actions). As new sensory evidence is accumulated and integrated with different representations of history-dependent action biases in different groups of PPC neurons, these multiple activity patterns may form a basis for changes in decision with accumulation of variable sensory evidence, favoring one neural group’s activity over another.

Decision making is complex and multifaceted, and the PPC represents only one of several brain areas that are collectively involved in its processing (Bucci, 2009). To date, most research regarding representations of decision making within the PPC have been related to perceptual decisions (such as motion discrimination tasks). However, motor behavior is not specific to such value-based decisions. An important area for further investigation is how the PPC signals relate to movement plans in response to social decisions for instance (Maddux et al., 2007). In other words, how are the complex emotions that arise in social settings represented within the PPC, and incorporated into movement plans such as reaching forward for a handshake, or turning away in avoidance behavior. Additionally, a representation for history-dependent action biases, and its incorporation into motor decision making implies a short timescale capability for learning and plasticity within this region of the brain (Rawley and Constantinidis, 2009; Seo et al., 2009). The malleability of the PPC to represent learning increases its appeal for its use in providing CNP control signals but requires investigation into the long-term performance of these CNPs.

Tremendous progress has been made in understanding the computational underpinnings of decision formation (for instance, the integration of sensory information leading to a decision) and their relation to neural patterns in multiple brain regions. An area that remains largely unexplored is the interaction between decision making processes in motor control with other processes, such as memory systems. Coordinated movements in the real world involve declarative memories about object properties (spatial memories about object properties, for example) and episodic memories about changes in the environment brought about by action. The PPC has been demonstrated to play a key role in memory-based navigation, and movement. Developing neural networks that can extract this complex interplay of data within this neuronal population may enable neuroprosthetics capable of movement but also of sensory adaptation, improved fluidity and dynamism through learning from prior errors, or memories, for instance. The shared neural resources within the PPC for the representation of memory systems, movement, and sensorimotor control now become reminiscent of shared computational resources through mixed selectivity models, and are an area in need of further exploration to eventually optimize CNP performance.

## Peripersonal Space

Our motor interactions within our sensory environment are often subconscious. Neuronal recordings in NHPs suggest that distributed cortical and subcortical networks encode an integrated neural representation of the body (“body schema”) and its immediate surroundings [“peripersonal space” (pPS), where objects can be grasped or manipulated, in contrast to “extrapersonal space,” where objects are not reachable without movement of the body or the objects] (Colby and Duhamel, 1991; di Pellegrino and Frassinetti, 2000; Sereno and Huang, 2014). The most widely studied of these neurons are simultaneously responsive to visual-tactile stimuli, although auditory-tactile and trimodal (visual-auditory-tactile) neurons have also been described (Graziano and Gross, 1993; Graziano et al., 1999). Critically, it has been demonstrated that the neural representation of the visuotactile pPS can be dynamically modified through experience, even on short timescales (Holmes and Spence, 2004; Holmes, 2012).

Visual-tactile bimodal neurons predominantly encode visually biased sensory schema (Graziano and Gross, 1993; Graziano, 1999). In a series of experiments with a taxidermied monkey hand, these neurons (in VIP and area 5d) responded to visuotactile stimuli applied to the dummy hand (with the real hand hidden from view) even when the dummy’s position conflicted with proprioceptive information regarding true hand position. In humans too, presenting ipsilesional visual stimuli to a dummy hand (with the real hand retracted from view) extinguishes contralesional tactile stimuli (Làdavas et al., 1998, 2000). Moreover, in humans, simultaneously viewing a dummy rubber hand being stroked by a paintbrush while experiencing similar stimuli on their own hand (hidden from view) altered the felt hand position (Farnè et al., 2003). These results imply that the brain’s representation of body schema and pPS is plastic. It can be altered by multisensory feedback and can allow the illusion of ownership of artificial but realistic effectors.

Plasticity in pPS representation has been explored extensively through tool-use (Azañón et al., 2010; Holmes, 2012). In crossmodal extinction studies in human subjects with parietal strokes, ipsilesional visual stimuli presented in the far extrapersonal space but at the end of a stick attached to the right hand extinguish an almost equal number of contralesional tactile stimuli as when presented to the hand in the near pPS (Làdavas et al., 1998; Farnè et al., 2003). This fails to remain true if the connection between the near and far spaces by way of the stick is removed. Moreover, in macaques, hand centered visual RFs of PPC bimodal neurons have been demonstrated to elongate after short periods of using a rake to retrieve food (Iriki et al., 1996; Serino et al., 2009). It is postulated that this results from the synchronous capture by PPC multimodal neurons of a tactile stimulus at the hand and an auditory (or visual) stimulus on the tool (in the extrapersonal space) (Rizzolatti et al., 1996, 1998; De haan et al., 2017). It remains to be elucidated whether this represents an updated postural model of the body in space (i.e., an incorporation of the tool into the body schema) or a remapping of extrapersonal visual space as pPS (Holmes, 2012).

Harnessing this plasticity in pPS representation may greatly enhance CNP functionality. The neural encoding of a body schema within the PPC reflects its position as an important node in motor control. Because of its direct connections with motor cortical areas such as M1, this may enable the rapid integration of sensory feedback with ongoing motor activity, improving coordination. From a BMI standpoint, a parietally driven CNP may benefit from this working model of the brain’s representation of body schema and pPS. However, the nature of the neural representation of an extracorporeal effector within the PPC remains unknown. Novel strategies to incorporate the plasticity into neuroprosthetics may include adaptive decoder designs that can integrate sensory schema with body schema. There remain several unanswered questions, such as for instance what the neural distinctions between representations of self, pPS and the new effector would be, whether sensory schema could be transferred to the CNP (as with the rubber hand experiment described above), and what the conditions necessary for the new limb to be incorporated into the body schema would be. Eventually answering these many questions may facilitate the development of a flexible, cognitive interaction of a parietally driven neural prosthetic with its environment.

## Conclusion

Brain machine interfaces and single neuron studies in human subjects and NHPs have vastly enriched our understanding of the brain’s complex neural networks and in particular, those of the PPC. Because of its strategic location, the PPC appears to be intimately involved in the processing of cognitive, higher level functions that are represented in its various sub-regions. We have briefly presented three of these in this report – representations of pPS, coordinate frame transformations, and movement related decision making. These are only a sampling but already begin to suggest that the various sub-regions of the PPC may be highly integrated in the processing and representations of several dimensionalities of movement that seamlessly work in concert to create coordinated dynamics. Understanding these higher-level representations may ultimately enable the most intuitive, flexible and versatile neural prosthetics with the greatest clinical utility.

## Author Contributions

SC was involved in the primary review of literature, the drafting of this manuscript in all its preparatory stages. TA, NY, and MJ contributed portions to the drafting of this manuscript and were instrumental in its review and improvement. NP provided invaluable guidance in the consolidation of ideas to be discussed in this manuscript and insightful reviews throughout its preparation.

## Conflict of Interest Statement

The authors declare that the research was conducted in the absence of any commercial or financial relationships that could be construed as a potential conflict of interest.
